# An alternative estimation of the death toll of the Covid-19 pandemic in India

**DOI:** 10.1371/journal.pone.0263187

**Published:** 2022-02-16

**Authors:** Christophe Z. Guilmoto

**Affiliations:** 1 Centre des Sciences Humaines, Delhi, India; 2 Ceped/IRD/Université de Paris/INSERM, Paris, France; Universidade Federal de Minas Gerais, BRAZIL

## Abstract

The absence of reliable registration of Covid-19 deaths in India has prevented proper assessment and monitoring of the coronavirus pandemic. In addition, India’s relatively young age structure tends to conceal the severity of Covid-19 mortality, which is concentrated in older age groups. In this paper, we present four different demographic samples of Indian populations for which we have information on both their demographic structures and death outcomes. We show that we can model the age distribution of Covid-19 mortality in India and use this modeling to estimate Covid-19 mortality in the country. Our findings point to a death toll of approximately 3.2–3.7 million persons by early November 2021. Once India’s age structure is factored in, these figures correspond to one of the most severe cases of Covid-19 mortality in the world. India has recorded after February 2021 the second outbreak of coronavirus that has affected the entire country. The accuracy of official statistics of Covid-19 mortality has been questioned, and the real number of Covid-19 deaths is thought to be several times higher than reported. In this paper, we assembled four independent population samples to model and estimate the level of Covid-19 mortality in India. We first used a population sample with the age and sex of Covid-19 victims to develop a Gompertz model of Covid-19 mortality in India. We applied and adjusted this mortality model on two other national population samples after factoring in the demographic characteristics of these samples. We finally derive from these samples the most reasonable estimate of Covid-19 mortality level in India and confirm this result using a fourth population sample. Our findings point to a death toll of about 3.2–3.7 million persons by late May 2021. This is by far the largest number of Covid-19 deaths in the world. Once standardized for age and sex structure, India’s Covid-19 mortality rate is above Brazil and the USA. Our analysis shows that existing population samples allow an alternative estimation of deaths due to Covid-19 in India. The results imply that only one out of 7–8 deaths appear to have been recorded as a Covid-19 death in India. The estimates also point to a very high Covid-19 mortality rate, which is even higher after age and sex standardization. The magnitude of the pandemic in India requires immediate attention. In the absence of effective remedies, this calls for a strong response based on a combination of non-pharmaceutical interventions and the scale-up of vaccination to make them accessible to all, with an improved surveillance system to monitor the progression of the pandemic and its spread across India’s regions and social groups.

## Introduction

India has been affected by the second wave of Covid-19 infections and deaths from February to September 2021. With the emergence of the Delta variant [1], this wave has been particularly severe and has hit most of the country. It spread in several urban agglomerations such as Mumbai, Delhi, Pune and Bengaluru which were among the hardest areas in the country and gradually to more rural areas [2]. However, there is a serious gap between the severity of the crisis reported by local communities and the effect measured by official indicators of contamination and Covid-19 related deaths. The country has officially registered only 459,000 Covid-19 deaths by early November 2021. This number corresponds to a rate of 0.3 per thousand inhabitants, which is twice lower than the world average [3]. Such a low figure contradicts the apparent severity of a crisis. The second wave struck most Indian families across the country and was accompanied by dramatic shortage of vaccines, Covid-19 tests, ambulances, access to health personnel, hospital beds, oxygen, ventilators, drugs and finally, coffins, wood, priests and spots for cremation or burials as widely reported in Indian and international media [4].

The purpose of this paper is to provide an alternative estimation method to compute the tally of Covid-19 deaths in India. We use here four independent population samples to reconstruct the impact of mortality. These samples of various sizes and demographic composition relate to official Covid-19 dataset for the state of Kerala, the employees of the Indian Railways, Indian elected representatives, and Karnataka’s schoolteachers. They are employed to characterize the age and sex profile of India’s Covid-19 mortality and then assess its severity by fitting mortality patterns to the number of deaths reported in these samples. This estimation implies a death tally of about 3.2–3.7 million from March 2020 to early November 2021, a figure about 7–8 times higher than the official numbers.

The paper starts with a brief presentation of the issues related to data quality and availability in India. The next section describes the alternative data sources on Covid-19 mortality and their characteristics. It also describes our estimation strategy based on the combination of mortality modeling and observed death rates. The final sections present the results of our estimation of Covid-19 deaths and a comparison of India’s death toll with the rest of the world. Methodological details are found in two separate appendices.

### Absence of reliable estimates of Covid-19 deaths in India

There are no direct estimates of deaths due to the pandemic in India except the official statistics that correspond only to a fraction of actual Covid-19 deaths. Cases of Covid-19 deaths are known to have been specifically underestimated for two main reasons: many deaths go unrecorded for lack of prior PCR test of the deceased; the cause of death is often selectively attributed to comorbidities (diabetes, asthma, etc.) and other apparent factors (heart attacks, etc.) when the WHO recommends suspected cases of Covid-19 to be reported as such [5–9]. More generally, Covid-19 is a source of shame. Families often prefer to hide the nature of the ailment affecting their relatives, ascribing resulting deaths to fevers or other diseases to avoid the social stigma [10–12]. This embarrassment is also felt at the political level, and Covid-19 figures have been used from the start of the crisis as a political chip to blame national and regional governments for wrong decisions or inaction or even to target minorities [13, 14]. This context provides no incentive at both micro and macro levels to record faithfully and exhaustively cases of Covid-19.

During the latest bout of mortality in March-May 2021, the press and civil organizations have actively reported the discrepancies between official figures of Covid-19 mortality and local evidence, resorting to innovative sources such as daily deaths counted in hospitals, a number of cremations and burials, corpses found floating in rivers, or obituaries published in local newspapers [15]. While highlighting the gaps in the death counts, this indirect evidence has not led to precise estimates at the aggregate level. No retrospective survey has been planned to estimate recent Covid-19 mortality [16, 17].

India’s civil registration is difficult to use for a variety of reasons. Causes of deaths are not adequately reported and estimates of the death toll can therefore only be drawn indirectly from registered deaths as excess mortality—which may be lower or larger than actual Covid-19 deaths due to the various effects of the lockdown and of the pandemic on other health conditions [18]. In addition, the quality of death registration greatly varies by region and between urban and rural areas [19]. Finally, death statistics are often published long after their occurrence with minimal information (if any) on the basic characteristics of deaths by age, sex, date, or cause.

The lack of reliable data has already led to alternative estimates. Early estimates are based on cross-regional modeling and “expert opinion” that are difficult to independently validate for India [20–22]. More recently, scholars have used available death registration figures to compute “excess mortality” (difference between expected and observed mortality) measurements from specific cities or regions. Some of these results will be discussed in the discussion below. The appraisal of the quality and representativeness of these different estimates would, however, require a separate review, which is beyond the scope of this paper. But the level of uncertainty of these estimates remains extreme as illustrated by recent estimates proposing ranges as considerable as 2.8–5.2 and 1.2–7.2 million deaths [23, 24].

### Alternative sources on Covid-19 mortality in India

The aim of this research is to number Covid-19 deaths since the beginning of the pandemic by identifying new sources on Covid-19 mortality from which to develop reliable estimates of deaths in India. The objective is not to compute Covid-19 excess mortality, which requires a complex set of hypotheses on expected mortality during a pandemic and representativeness of existing figures. In this paper, we have tried to evaluate the number of deaths directly attributable to Covid-19, but we could not assess the overall impact of the pandemic on Indian mortality.

Several sources provide the number of Covid-19 deaths for specific communities, organizations, or localities. For this purpose, we have examined most of the available samples of mortality statistics related to COVID-19 and verified their reliability, completeness, and representativeness. We have also focused on the entire pre-vaccination period (from February 2020 to May 2021) as samples might have not been affected uniformly by the vaccination drive. The selection criteria were the following:

1. Samples based on well-circumscribed populations (denominator).
2. Reliable estimation of the Covid-19 deaths (numerator)
3. Samples large enough for mortality estimates (robustness).
4. Populations with information on their age and sex structures (demographic composition)
5. Samples covering the entire pre-vaccination period from March 2020 to May 2021.
6. Samples representative at all-India level (coverage).

After examination, we discarded most sources that provided incomplete or potentially unrepresentative information. The following samples were rejected:

- **Indian Armed Forces** (119 deaths). It is a large sample, composed mostly of young men below 40, but we cannot use it for lack of detailed information on its demographic composition.
- **Medical community** (244 deaths among doctors during the second wave). It is a small sample, but it was rejected chiefly due to its extreme exposure to contamination during the pandemic. The same is true for several other samples of health personnel.
- **Schoolteachers in Uttar Pradesh** (1621 deaths). We discarded this sample for several reasons: lack of demographic characteristics of the population at risk, lack of data for 2020, and suspiciously high mortality rate in 2021.
- **Bank employees of India** (1,300 deaths). This sample is not usable due to the lack of basic information on this sample’s demographic characteristics.
- **Employees of Hindustan Aeronautics Limited** (100 deaths) and **civil aircraft pilots** (17 deaths). These samples are too small and undocumented.
- **Death claims from insurance companies** (35,500 deaths). This sample is incomplete in terms of deaths, and we lack the necessary information on its demographic composition. In addition, the insured population may not be representative of India’s population.
- **Observed deaths in specific municipalities/states**. Issues of representativeness and interpretation related to excess mortality computed from civil registration statistics would require a separate study.

We finally retained the following four samples, which met our criteria in terms of reliability of death estimation, regional representativeness, and demographic characteristics.

- **Kerala sample**: Dataset of deaths by age and sex in Kerala state
- **MLA sample**: Deaths of elected representatives
- **IR sample**: Deaths of Indian Railways personnel
- **Karnataka sample**: Deaths of schoolteachers in Karnataka state

It should be kept in mind that it remains impossible at this stage to formally test the reliability of the last two sources for lack of disaggregated datasets and the absence of additional information on their mortality experience. In the following paragraphs, we describe the main characteristics of these samples and discuss their representativeness for estimating India’s Covid-19 mortality.

A. The **Kerala sample** is a dataset with 26,628 Covid-19 deaths classified by age and sex on 18 October 2021. The death toll has been compiled and regularly updated by the bureau of statistics of the State of Kerala. It is available online as a database of individual deaths with their date and place of occurrence and the age and sex of the deceased [25].

This data source is unique for India. To the best of our knowledge, no other statistical office at the regional or national level has ever published in the country disaggregated information on Covid-19 deaths in an accessible format. It comes from a strong public health system, whose response to the outbreak has been internationally lauded [26]. Kerala is also known for the quality of its demographic information, a feature related to its overall educational level. While not perfect, death registration is considered nearly complete in Kerala [27]. This dataset still reflects some amount of age heaping for ages ending with 0 and 5 and we computed the mortality rates after smoothing the age at Covid-19 deaths with a moving average over five successive years. However, death rates computed on the smoothed age distribution are almost identical to rates computed on the raw data.

The Kerala sample will be used only to model the age and sex schedules of Covid-19 mortality in India since it is the only source of Covid-19 casualties given by sex, age, and date of death. A more detailed discussion of this age and sex schedule is found in S1 Appendix (Modeling and standardizing Covid-19 death rates by age).

B. The **MLA sample** is relatively small sample of 5,837 individuals. Individuals in this sample are the elected representatives of India, starting with the MLAs (Members of the Legislative Assembly) sitting in the regional *Vidhan Sabha*s. We have added members of the national parliament, i.e., the *Lok Sabha* (534 members) and the *Rajya Sabha* (226 members). MLAs represent 85% of the entire sample. About 0.5% representatives are missing from the sample due to lack of information (or vacant seats). Elected representatives cover the entire territory of India, even with 5 smaller territories without legislative assemblies.

The detailed age and sex data of this population are available from published databases such as Trivedi Centre of Ashoka University and the websites of the Lok Sabha and the Rajya Sabha [28–30]. The overall age distribution is applied to the part of the sample (20%) for which age information is missing from existing sources. Women account for only 7.1% of representatives. The mean ages are relatively high at 56.2 years for men and 50.4 for women.

Deaths by Covid-19 of elected representatives have been systematically covered by the national or regional press, with precise information on the date and location of the event. Deaths of elected representatives also lead to vacancies or by-elections reported in the media. We used a list of Covid-19 deaths in India to crosscheck our results. We conducted a systematic search of deaths attributed to Covid-19 of the MLA sample from 2020 to 25 May 2021 using press reports.

**Fig 1:**
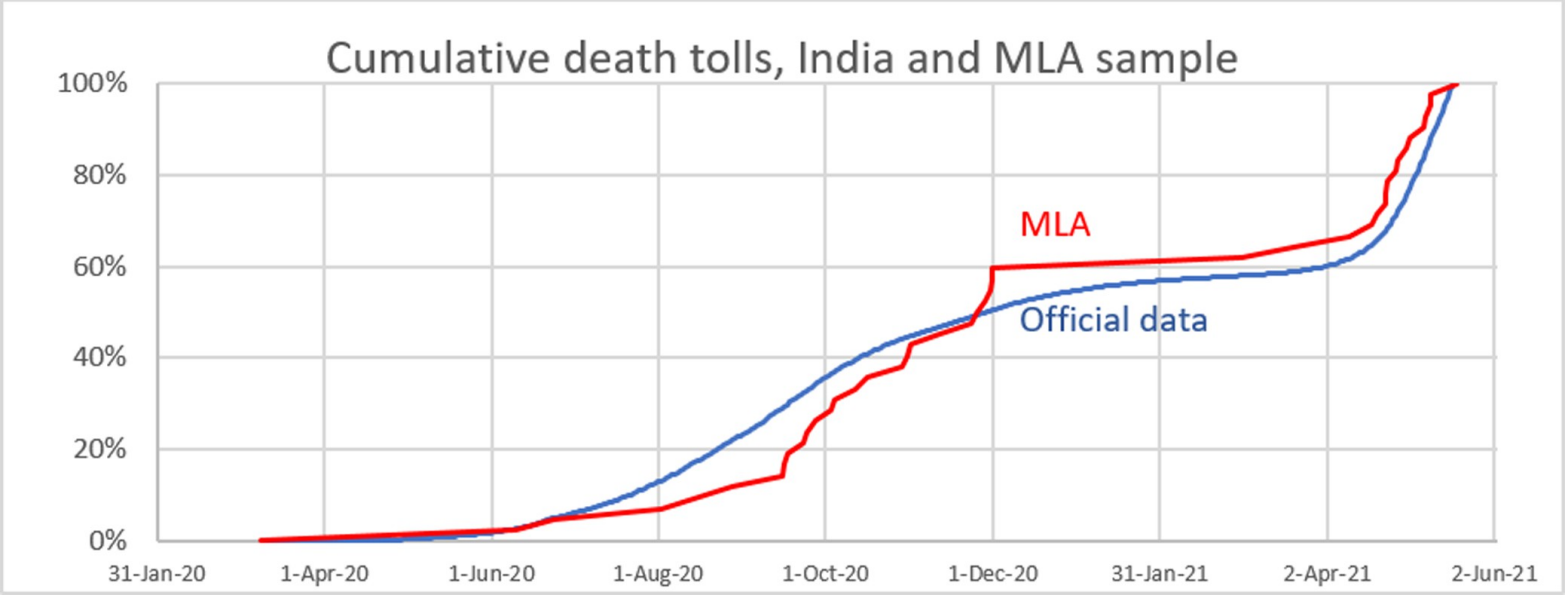
Cumulative Covid-19 deaths from February 2020 to 20 May 2020, Indian official estimates and MLA sample percentage of total deaths). Source: computed by the author.

A total of 43 deaths by Covid-19 of elected representatives were reported, which points to a rather high fatality rate of 74 per 1000 in this subpopulation. The average age at death in the MLA is 65.6 years. Fig 1 shows that the cumulative distribution of deaths since March 2020 in the MLA sample reflects the officially reported Covid-19 deaths in India. 60% of these deaths occurred during the first wave (i.e., before February 2021) as against 57% according to the official estimates of Covid-19 deaths for India.

This is an admittedly small sample but of high quality and precision. The MLA sample corresponds to a high stratum of Indian society and represents one of India’s most visible elite groups. They are primarily city-based, and apart from their relatively high salaries, they enjoy numerous added benefits such as housing, home staff, specific allowances, traveling and energy reimbursement, access to medical facilities, and pension benefits. This situation was reflected notably by the fact that a vast majority of Covid-19-related deaths among MLAs took place in the most reputed Indian hospitals across the country. Keeping in mind that survival in India is closely linked to socioeconomic status [31, 32], elected representatives have most likely suffered far less than the rest of the population.

This elite population was subjected to a high level of exposure to contamination with the population of their constituencies. Many politicians initially expressed support for unproven local remedies and did not follow standard protective measures based on social distancing. Like politicians elsewhere in the world, they continued to meet hundreds of people on a regular basis in meetings of various sizes and many got contaminated early on during the pandemic. The activities of regional and national representatives in India never stopped throughout the pandemic, especially during the different elections rounds in November 2020 and April 2021 in five states and territories and the local (*panchayat*) elections held in India’s largest state in April 2021 during the middle of the second wave. These elections (and a major pilgrimage held at the same period) were in fact, blamed for the accelerated diffusion of the coronavirus in North India during the second wave of 2021.

In conclusion, we consider that the high exposure level of this subpopulation has probably offset their socioeconomic advantages, and the mortality level derived from the MLA sample should be rather close to the Indian average situation.

C. The **IR sample** (where IR stands for Indian Railways) is a large sample that relates to the employees of the Indian Railways. With 1.3 million employees in 2020, the Indian Railways is, in fact, one of the world’s biggest employers [33]. The IR network covers the entire territory of the country, except for insular and mountainous regions. It announced on May 10 that 1,952 staff died of Covid-19 (1.5 per 1000), a figure that should refer to the previous Friday (May 7) at the latest [34]. For comparative purposes, the total will be projected until May 25 in proportion to the rise of official Covid-19 deaths for a comparison with the MLA sample.

Covid-19 deaths are likely to be adequately numbered for two main reasons. First, the IR is an organization with a unique internal health system (the Indian Railway Health Service with 13,600 hospital beds, 2,600 doctors, and 41,000 other health staff), through which employees and their families enjoy access to quality healthcare, including in time of Covid-19 crisis. Second, the main union of the Indian Railways has long been calling for a Covid-19 higher compensation for deceased employees, a move that has probably encouraged families to adequately report cases of Covid-19 deaths.

Like most Indian organizations, the IR does not publish statistics on the demographic composition of its workforce. The only information included in its regular yearbooks is the share of female employees, standing at 99 thousand workers in 2020 (7.9%). We have enough information to reconstruct the age structures of its workforce by using the average age of employees (45 years) and the share of employees aged 50 or above (40%) [35, 36]. Using these two parameters, we have fitted a linear distribution by five age groups from ages 20 to 60 (ages for recruitment and retirement). Non-linear age distributions with similar demographic characteristics have been tested with no significant bearing on mortality estimation.

The IR sample represents a share of India’s workforce that is quite heterogeneous and spread across regions. It includes all socioeconomic groups (from peons to engineers) and covers all social groups, including lower-status groups (Other Backward Classes, Dalits, and Adivasis) who enjoy job reservations. Overall, IR workers may be slightly more privileged than the rest of the population for various reasons: it is a more urban population (following the railway grid) made of permanent employees (public servants) that have in particular access to income security, housing allotment or subsidies, dedicated healthcare system, provident fund, and pension benefits. By definition, this sample does not cover workers from the informal economy. It is also a population that may have been exposed to higher risks of Covid-19 contamination due to the volume of passenger traffic (8.1 billion in 2019–20). As proof, the death rate is twice as high among station masters (2.9 per 1000) as among the rest of the IR staff (1.5 per 1000). But most of the IR staff are not frontline workers but engineers, clerks, mechanics, pilots, and technicians (such as *pointsmen* or *gangmen)* who are not in contact with the public. The IR traffic has also been repeatedly interrupted during the pandemic, most notably during the initial lockdown in March-May 2020, during which almost no train ran through India. However, the vigorous vaccination campaign that started in May 2021 among the IR staff has probably reduced subsequent Covid-19 mortality in a significant way [37].

For all these reasons linked both to its social and regional composition and limited exposure levels to contamination, this subpopulation represents a slightly privileged part of the workforce due to its employment status and attached benefits. Their mortality level should represent the lower bound of our estimates.

D. The **Karnataka sample** is a sample of 268 deaths attributed to Covid-19 among schoolteachers of the southern state of Karnataka. This number of deaths was reported in the press on May 13. Albeit small, this number can be related to the demographic structure of Karnataka’s 196,163 teachers. The sex and school-level composition of teachers in Karnataka can be found in the most recent administrative report [38]. Unlike the MLA and IR samples, a majority of them are women (52.6%). In addition, a special study provides an estimate of the average age of the schoolteachers (39.5 years) [39]. This number closely corresponds to the official age limit of the profession (from 21 to 59 years). While teachers may represent vulnerable frontline workers, schools in Karnataka were closed for most of the pandemic period and may not have been more exposed to the virus than the general population.

Karnataka apparently recorded a larger proportion of cases and deaths than the rest of India, with the metropolitan region of Bengaluru severely hit by both waves of the pandemic. For this reason, this sample cannot be used to estimate the national mortality level. However, it will be used to test the validity of mortality estimates derived from the IR sample.

### Mortality estimation strategy

The methodology used for estimating mortality in India is as follows.

We first use Kerala data to determine the age and sex schedule of Covid-19 mortality in India, but not to estimate its intensity. We apply the Kerala Covid-19 mortality rates to project the number of deaths in the three other samples (MLA, IR and Karnataka). We then adjust the intensity of mortality to fit the number of observed deaths in these three samples. These adjusted mortality patterns can be applied to India’s age and sex structure to yield national-level estimates of Covid-19 mortality in May 2021. The numbers of deaths are finally projected to 15 October 2021 using trends reflected in the official Covid-19 casualty statistics and contrasted with international figures using standardized rates.

The estimation procedure rests on two assumptions. The first hypothesis posits that these samples reflect the severity of Covid-19 mortality in India. As already discussed, we consider the IR and MLA samples close to the average Indian mortality experience even if they still represent lower-bound values in view of their privileged status.

The second hypothesis posits that Kerala can be used as the prototype of India’s age and sex patterns of Covid-19 mortality. We can then adjust the intensity of mortality according to a Covid-19 mortality model based on previous studies on the age and sex distribution of Covid-19 mortality across the world.

In relation to the second hypothesis, S1 Appendix discusses in detail the quality and applicability of Kerala’s Covid-19 death rates by age and sex. We will, however, briefly introduce the readers to the main argument of the reasoning: Covid-19 mortality by sex and age has now been measured in most countries in the world with disaggregated statistics published by national statistical bureaus [40]. Beyond the overall observation that Covid-19 mortality rises with age and is more pronounced among males, the comparison of these mortality curves has highlighted their extreme regularity. Covid-19 mortality above age 20 follows the so-called Gompertz law of mortality (in which death rates can be expressed as an exponential function of age) [41–43]. As a result of this mathematical regularity, death rates unknown at one given age can be extrapolated from death rates observed at other ages. Another advantage of the Gompertz modeling is its partition of mortality into two main components:

- Its age distribution (i.e., the slope or the *tempo* of mortality) corresponds to variations by age
- Its intensity (the level or *quantum* of mortality) captures the overall severity at all ages.

The age profile of Kerala’s Covid-19 death rates is perfectly in line with mortality patterns observed elsewhere, notably with patterns measured in its neighbor Sri Lanka (S1 Appendix). We can subsequently use Kerala’s rates to simulate mortality in other samples (IR, MLA, and Karnataka samples) by adjusting the quantum of mortality to deaths observed in other samples, keeping age patterns (the tempo) fixed.

Once we have estimated the parameters of Covid-19 mortality corresponding to each of these samples, we can apply them to India’s 2021 population and compute the number of Covid-19 deaths till 1 November 2021, at the end of the second wave. For the final discussion, we have added comparative data from the USA and Brazil and computed a comparative standardized Covid-19 death rate.

The different steps in the estimation procedure can be summarized in five steps.

1. We first compute age and sex Covid-19 death rates based on the Kerala sample and then derive from these rates a preliminary Gompertz model of Covid-19 mortality.
2. This model is applied to the samples’ populations classified by sex and age and yields the hypothetical Covid-19 death tolls if the Kerala Covid-19 mortality had been observed.
3. The intensity of the model then adjusted to fit the number of deaths observed in these samples.
4. The resulting adjusted mortality model is applied to India’s population by age and sex, yielding the projected national number of Covid-19 deaths.
5. India’s Covid-19 mortality is projected to November 2021 and its intensity compared with deaths rates from Brazil, the USA and the world.

**Fig 2:**
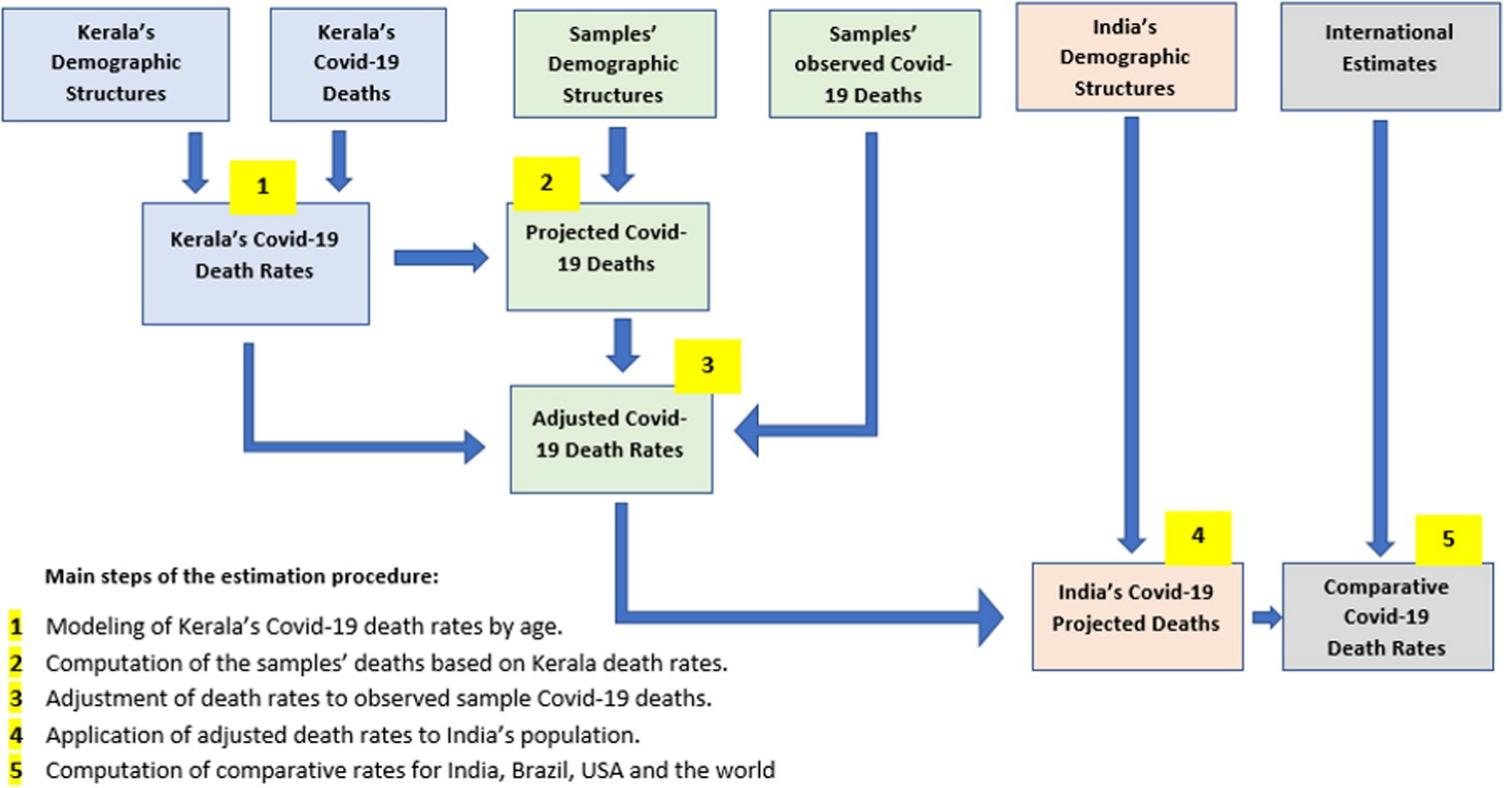
Procedure for estimating India’s Covid-19 deaths.

The first four steps of the workflow are summarized in Fig 2. Further explanations and illustrations are found in S1 and S2 Appendices.

## Findings

Table 1 summarizes the results of the estimation procedures applied to the IR and MLA samples. The estimated total number of Covid-19 deaths in India was 1.7 million till 7 May 2021 (IR sample) and 2.6 million till 25 May 2021 (MLA sample). For the latter date, the estimates range from 2.3 (IR sample) to 2.6 million (IR sample). These estimates are 7.0 and 8.6 times greater than official estimates respectively. The projected death toll is 3.2–3.7 million by 1 November 2021.

**Table 1:** Estimates of cumulative Covid-19 deaths in India.

	Sources			Units	Reference date (in 2021)
	Official estimate*	IR Sample	MLA sample		
**Covid-19 deaths**	243.5	1,701.8	**-**	000s	7 May
	325.8	2,276.6**	2,597.1	000s	25 May
	458.9	3,206.9**	3,658.3**	000s	1 November
**Correction factor**	1.0	7.0	8.6	***	1 November
**Death rates**	0.3	2.3	2.6	per 1000	1 November

*: Smoothed cumulative series from Worldometer [3].

**: Projected using the trends in official Covid-19 deaths.

***: Computed as estimated deaths/official deaths.

There are variations in our estimates as the MLA sample shows death figures that are 14.1% higher than with the IR sample. This difference may reflect measurement issues as well as the composition of each sample. Higher mortality observed among elected representatives (MLA sample) may be attributed to their exposure to contamination since the outbreak of the pandemic due to their continuous interaction with their electorate and the busy election schedule of 2020–21. In comparison, the mortality estimate based on the IR staff may capture a less exposed subpopulation even if frontline workers. The IR estimate gets a first confirmation from the Karnataka sample. This sample recorded 268 Covid-19 deaths among government schoolteachers in the Southern state of Karnataka. The application of the IR mortality patterns to the age and sex structure of Karnataka’s schoolteachers yield a preliminary total of 177 deaths. When we correct the latter figure for Karnataka’s relatively higher Covid-19 death rate (66% above India’s as per official estimates in May 2021), we obtain a final estimate of 294 deaths among schoolteachers based on the IR mortality level. This figure is very close to the 268 deaths reported among Karnataka’s schoolteachers and reinforces the consistence of the figures based on the IR sample.

Considering all these observations, we keep 3.2–3.7 million as our best estimates by 1 November 2021. This probably represents a lower bound value since our main samples do not cover the most underprivileged sections of India’s population, notably rural and informal workers.

## Discussion

All over the world, the identification and proper registration of Covid-19 deaths have been a challenge. Due to data restriction or severe underestimation in many countries, Covid-19 mortality has most probably also been severely underestimated in Brazil, Iran, Peru, Russia, or Turkey [44–48]. In India’s case, the total number of deaths till November 2021 is estimated at 3.2–3.7 million at the end of India’s second wave. These figures are higher than the official death toll by a factor of 7–8. This pronounced undercount is the joint product of the frequent under-registration of adult deaths in India, the reluctance or inability of families and local authorities to correctly identify Covid-19 deaths, and the faulty attribution of Covid-19 deaths to other causes.

Our estimates appear comparable to other estimates of excess mortality that have appeared in working papers and preprints [23, 49–51]. The latter studies provide estimated figures extrapolated from existing civil registration statistics of 3.4, 2.7, 3.8, and 3.2 million excess deaths respectively. These provisional estimates average at 3.3 million, a level that coincides with our estimates of 3.2–3.7 million Covid-19 deaths until November 2021.

The salience of these estimates appears more clearly when set against figures from other countries (Table 2). With 3.2–3.7 million Covid-19 deaths by November 2021, India now emerges as the country with by far the largest number of deaths in the world, well ahead of the USA (0.8 million), Brazil (0.6), or Mexico (0.3). If India’s revised estimates were incorporated, the world’s death toll would rise by several million to 7.8–8.3 million Covid-19 deaths till 1 November 2021.

**Table 2:** Comparative indicators of Covid-19 mortality.

	Units	India (IR-MLA samples)	Brazil	USA	World
Population *	million	1393.0	214.6	333.6	7,875
Average age*	years	31.0	35.1	39.5	33.2
Total Covid-19 deaths**	million	3.2–3.7	0.6	0.8	5.0
Covid-19 death rate**	per 1000	2.3–2.6	2.8	2.3	0.6
Standardized Covid-19 death rate***	per 1000	3.0–3.4	2.7	1.3	0.6

Sources: *demographic data from United Nations estimates (World Population Prospects 2019) [52];

**Computed from national estimates of deaths in October 2021 by age and sex [53, 54] (our estimates for India) and United Nations estimates [52].

An important result of our estimation relates to Covid-19 mortality rates. The highest crude Covid-19 death rates per inhabitant in November 2021 are observed in Peru with 6.0 per 1000 and in Eastern Europe where several countries record rates above 3.0 per 1000. India’s revised Covid-19 death rate of 2.3–2.6 per 1000 is four times as high as the world’s average (0.6) and would place India at the 13-19^th^ rank of most affected countries—rather than at the 127^th^ rank per official estimates.

In addition, as detailed in detailed in the discussion on the age factor has detailed, these crude Covid-19 death rates are directly affected by the age and sex composition of each population [41, 55]. We have therefore computed age and sex-standardized death rates by applying observed national death rates to the world’s population in 2020 (see methodology in S1 Appendix and illustration in S2 Appendix). Once corrected for its specific demographic structures, India’s standardized Covid-19 mortality rate now stands at 3.0–3.4 per 1000 inhabitants. This standardized rate is above Brazil’s and 5 times as high as the US rate. This standardized rate—which reflects the severity of the crisis for all age groups rather than its overall mortality impact, is clearly among the world’s highest.

The true extent of the coronavirus outbreak in India requires immediate attention and support from the international community to help Indian authorities respond to all aspects of this health crisis by combining non-pharmaceutical interventions and scale-up of vaccination to make them accessible to all. This study chiefly emphasizes the need for an improved surveillance system to monitor the evolution of the pandemic. The lack of proper statistics and India’s relatively favorable age structure have concealed the extreme severity of India’s Covid-19 crisis.

As a final observation, we should stress the limitations of our approach. The estimates presented here remain intricately linked to the quality of information in the population samples. In the future, large new samples should ideally include vulnerable subpopulations underrepresented here such as womenfolk, urban migrants, the peasantry and informal workers, and the slum population. They may also allow better document the poorly understood socioeconomic and gender differences in Covid-19 mortality. In addition, many questions remain unanswered such as the breakdown of Covid-19 deaths by sex or region, the specific impact of the second wave and of the new Delta variant originating from India, and the contribution of local comorbidity pattern to the death toll.

## Supporting information

S1 Appendix Modeling and standardizing Covid-19 death rates by age. (DOCX)

S2 Appendix Computations. (DOCX)

S1 Table Population, Covid-19 deaths and Covid-19 death rates, Kerala, India, 2021. (DOCX)
