# Supplementary material for: An alternative estimation of the death toll of the Covid-19 pandemic in India
Source: PLoS One. 2022 Feb 16;17(2):e0263187. doi: 10.1371/journal.pone.0263187 (PMC8849468; doi:10.1371/journal.pone.0263187)
Supplement: S1 Appendix — (DOCX) [file pone.0263187.s001.docx]

**An alternative estimation of the death toll of the Covid-19 pandemic in India**

Christophe Z Guilmoto

Centre des Sciences Humaines, Delhi

Ceped/IRD/Université de Paris/INSERM, Paris

**Appendix 1**

# Modeling and standardizing Covid-19 death rates by age

The distribution of Covid-19 deaths by age and sex is crucial to understand and model the impact of the pandemic. In this appendix, we examine the disaggregated mortality data of India’s state of Kerala

## The age factor of Covid-19 mortality

In countries with better statistical systems, Covid-19 cases are tabulated by age group, and disaggregated data have allowed for the computation and analysis of Covid-19 death rates by age and sex in various countries [1-3]. This led to the recognition of the strong age gradient of Covid-19 mortality observed throughout the world. Research on Covid-19 mortality has shown that male rates are systematically higher than female rates, and the progression of mortality with age is very rapid, faster than for ordinary mortality. For instance, Covid-19 mortality risk increases tenfold when age increases by 20 years. This means that Covid-19 mortality risk is ten times as high at 65 as 45. The age composition of the population at risk plays a crucial role in the overall death toll, a feature already illustrated by the high Covid-19 death rates observed in North America and European countries.

The regularity of mortality patterns has also highlighted that Covid-19 mortality follows the typical features of the so-called Gompertz law [4]. In this classical mortality model, age-specific mortality rates can be approximated as an exponential function of age. The standard Gompertz equation is:

(1) d(*x*)= e *^Ax + B^*

where d(*x*) is the death rate at age *x*, while A and B are parameters.

Coefficient A (>0) captures the age distribution of mortality, i.e., the slope of the curve (the *tempo* of mortality), while coefficient B corresponds to the overall intensity (the *quantum* of mortality) and affects mortality levels at all ages. These two coefficients allow for a complete parameterization of age-specific mortality rates according to their tempo (early vs. late mortality) and quantum (low vs. high mortality). This type of modeling is therefore applicable to Covid-19 mortality rates by age.

## Comparing Kerala’s series of age-specific death rates with other series

We start with the dataset of Covid-19 deaths published regularly by Kerala’s statistical office [5]. It corresponds to 26,628 Covid-19 deaths up to 18 October 2021. We now use this series to compute the age - and sex-specific Covid-19 mortality rates by computing the death ratio per person. In the absence of 2021 census figures, we use Kerala's projected age and sex distribution estimated for 2021 [6]. All data and computations are provided as supplementary Table (S3: Population, Covid-19 deaths and Covid-19 death rates, Kerala, India, 2021). The death rates for men and women are shown below in Figure A1.

The series display a regular increase of death rates from 10-15 to 85+ and male rates distinctly higher than female rates (note the log scale used here). Death rates seem relatively erratic for ages below 10 due to the small number of reported deaths. There are for instance only one Covid-19 death among girls aged 5-9 years. Death rates at younger ages are almost negligible and do not influence the overall Covid-19 death toll. This series cannot be used to summarize India’s overall mortality level (which may be higher or lower than Kerala’s), but it will be used to model death rates by age in India in the absence of any other detailed Covid-19 statistics for the rest of the country.

For comparative purposes, we assembled a set of four series of death rates by age and sex, starting with Kerala’s death rates based on 26,628 deaths recorded on 18 October. We also computed the age-specific Covid-19 death rates based on available cumulative data of deaths by age for Brazil (592,178 deaths on 15 October 2021) and the USA (734,515 deaths on 13 October 2021), the two countries with the largest number of Covid-19 casualties in the world [7-8].We added to our comparison the only South Asian country to publish detailed Covid-19 deaths by age and sex, viz Sri Lanka [0]. Sri Lanka experienced a severe third wave peaking in August 2021, and we used the 13,267 Covid-19 deaths reported on 18 October 2021. The death rates for these three countries are computed based on the 2020 age and sex structure estimated by the United Nations in 2019.

**Figure A1: Covid-19 death rates by age and sex, Brazil, Kerala, Sri Lanka, and the USA, October 2021.**

**Source:** computed by the author from [5, 7-9]

Figure A1 illustrates several Covid-19 mortality profiles across the world. We see strong exponential relation between age and Covid-19 death rates in each country and the parallel profile of all mortality patterns. Brazil records the death rates at all ages followed by the USA. Kerala’s profile is distinctly below those of Brazil and the USA. Interestingly, the distribution of death rates by age in Kerala is also almost identical to Sri Lanka’s, with only slightly lower Covid-19 death rates in the latter.

Further analysis also shows that the progression of Covid-19 death rates tends to decelerate above age 70 in developing countries [4, 10]. This feature is noticeable for Kerala and Sri Lanka and gives the death curve a slightly concave aspect at older ages. There is also a Covid-19 mortality surge below age 5. This feature mirrors the cusp of general mortality, which is invariably higher at ages 0-4 than at ages 5-9.

## Modeling the age distribution of COVID-19 deaths by sex based on Kerala’s series

This comparison shows that death rates computed on the Kerala sample follow the expected Gompertz profile and are parallel to patterns observed in Sri Lanka in terms of both slope (tempo) and intensity (quantum) of mortality. Going beyond the linear model used for adult mortality, we have introduced additional parameters to adjust the model of mortality by age to fit the situation of the younger and older age groups already noted. To better model Kerala Covid-19 mortality schedule, we use a more complex equation with four parameters:

(2) d(*x*)= e *^Ax³ + Bx² +Cx +D^*

where d(*x*) is the Covid-19 death rate at age *x* and *A, B, C*, and *D* adjustment parameters. Parameters A and B account for mortality above 70 and below 10 respectively. Parameters *C* and *D* capture the overall age schedule (tempo) and the overall quantum of Covid-19 mortality respectively. This parameterization provides a better fit for the turnaround of Covid-19 mortality at young ages and its deceleration at older ages (Figure A2).

**Figure A2: Observed and modeled Covid-19 death rates (log) by age and sex, Kerala, 18 October 2021.**

**Source:** computed by the author from [5]

This model provides an almost perfect fit for adult ages, with r²= .997 (males and females). It is less precise for children because of the near absence of cases of child deaths and the cusp observed at ages 5-9 years. The overall correlation remains extremely strong with r² at .98 for male rates and .94 for female rates and the gap for death rates below age 15 has almost no repercussion on the overall mortality toll due to the extremely low level of mortality risk at young ages (death rates <5 per million for the population aged less than 10).

The advantages of this generic model of Covid-19 mortality by age and sex for our estimation procedures are twofold:

- The model allows for the estimation of Covid-19 death rates at all ages even when death rates may be missing for age groups thanks to the coefficients *A, B*, and *C*.
- The model can be adjusted to fit different overall mortality levels with the help of coefficient *D*.

## Standardizing national estimates of COVID-19 deaths

Crude Covid-19 death rates observed in a given country are directly affected by its age and sex composition. Countries such as the USA with a larger proportion of population aged 60+ are most vulnerable to Covid-19 mortality while younger countries Sub-Saharan Africa will be the least vulnerable. Therefore, they will register more deaths than relatively younger countries like Brazil or India, *ceteris paribus*. To offset the effect of demographic structures, we also have standardized Covid-19 death rates. The standardization procedure consists of the application of each country's observed death rates by age and sex to a fixed age and sex structure. We use for this purpose the age and sex distribution of the world’s population in 2020 as projected by the United Nations in 2019.

We use the death rates by age and sex computed previously for Brazil and the USA in mid-October 2021 and the death rates derived for India from both the IR and MLA sample and updated to 1 November 2021. These standardized death rates are not any longer affected by each country’s demographic structures. They reflect the severity of COVID-19 mortality irrespective of the age distribution of the population.

An alternative method of standardization (not followed here) uses US Covid-19 death rates by age and sex to correct the impact of national or subnational population structures on observed COVID-19 mortality [11]. It may not be applicable to the case of countries such as India where the Covid-19 mortality at old ages differs from that of the USA (Figure A1).

**References:**

1. The demography of Covid-19 deaths. 2021 [cited 2022 Jan 19]. Ined, Paris. Database available from: https://dc-covid.site.ined.fr/en/data/
2. Demographic Observatory Latin America and the Caribbean 2020: COVID-19 mortality. Evidence and scenarios. CEPAL. 2020 [cited 2022 Jan 19] Available from: https://repositorio.cepal.org/handle/11362/46641
3. Bauer P, Brugger J, Koenig F, Posch M. An international comparison of age and sex dependency of COVID-19 Deaths in 2020 - a descriptive analysis. Sci Rep. 2021. 11; 19143. Available from: <https://doi.org/10.1038/s41598-021-97711-8>
4. Guilmoto CZ. COVID-19 death rates by age and sex and the resulting mortality vulnerability of countries and regions in the world. MedRxiv. 2020 May 20 [cited 2022 Jan 19]. Available from: doi: https://doi.org/10.1101/2020.05.17.20097410
5. Government of Kerala. State Dashboard [cited 2022 Jan 19]. Database available from: https://dashboard.kerala.gov.in/
6. National Commission on Population, Population Projections For India And States 2011–2036 Report Of The Technical Group On Population Projections, Ministry of Health and Family Welfare. 2019 November
7. Óbitos com Suspeita ou Confirmação de COVID-19. Cartórios de Registro Civil do Brasil, [cited 2022 Jan 19]. Database available from: https://transparencia.registrocivil.org.br/especial-covid
8. Provisional Covid-19 Deaths by Sex and Age. Center for Disease Control and Prevention [cited 2022 Jan 19]. Database available from: https://data.cdc.gov/NCHS/Provisional-COVID-19-Death-Counts-by-Sex-Age-and-S/9bhg-hcku
9. Epidemiology Unit [cited 2022 Jan 19]. Ministry of Health, Colombo. Database available from: http://www.epid.gov.lk/web/index.php?option=com_content&view=article&id=233&lang=en
10. Demombynes G. et al. COVID-19 age-mortality curves for 2020 are flatter in developing countries using both official death counts and excess deaths, working paper, World Bank, Washington, DC, 2021.
11. Heuveline P, Tzen M. Beyond deaths per capita: comparative COVID-19 mortality indicators. BMJ open. 2021 Mar 1;11(3):e042934. Available from: https://bmjopen.bmj.com/content/11/3/e042934
