# Supplementary material for: An alternative estimation of the death toll of the Covid-19 pandemic in India
Source: PLoS One. 2022 Feb 16;17(2):e0263187. doi: 10.1371/journal.pone.0263187 (PMC8849468; doi:10.1371/journal.pone.0263187)
Supplement: S2 Appendix — (DOCX) [file pone.0263187.s002.docx]

**An alternative estimation of the death toll of the Covid-19 pandemic in India**

Christophe Z Guilmoto

Centre des Sciences Humaines, Delhi

Ceped/IRD/Université de Paris/INSERM, Paris

**Appendix 2**

# Computations

In this section, we illustrate the estimation procedures with the case of the IR sample.

We first distribute the sample population by sex and age according to the available information. In the case of the IR sample, we use four types of information: the population size, the share of women, the average age of the workforce, and the proportion among them aged 50+. To conform to general age regulations pertaining to recruitment and retirement in the Indian railways, the sample population is strictly contained in the 20-59 age band. We use here a simple linear distribution by age, but other imputations do not affect the results. The imputed population distribution is shown in columns 2-3 of Table A1.

We then apply mortality rates by age and sex derived based on the Kerala model (see Appendix 2) shown in columns 4-5 of Table A1, with the number of projected deaths shown below. The death by age and sex is then adjusted upward (columns 6-7) to match the observed death rates reported by the Indian Railways (i.e., a total of 1,952 deaths).

We finally applied the revised model extended to all ages from 0-4 to 80+ to the entire age and sex structure of India. India’s figures shown in columns 8-9 are drawn from the United Nations estimates published in 2019 (World Population Prospects). This simulation yields a total number of 1.65 million deaths up to May 7 (columns 10-11). For comparative purposes, this total will be projected to 2.16 million on May 25 using the observed of official Covid-19 deaths during this period (+30.7%).

We finally use the world age and sex structure derived from the United Nations estimates to standardize the Covid-19 death rates. To do that, we apply the world’s sex and age structure in 2020 to India’s population total (columns 12-13). We finally apply the revised mortality model (columns 6-7) to India’s standardized age and sex distribution (columns 12-13) to get the standardized deaths (columns 14-15), yielding the standardized Covid-19 death rate for India (lower row). The estimates refer to May 2021.

The other computations derived for the MLA and Karnataka samples follow an identical pattern.

**Table A1: Estimation procedure of Covid-19 mortality in India based on the IR sample (May 7, 2020).**

|  | **Indian Railways workforce** | | **Kerala Covid-19 death rates** | | **Adjusted death rates** | | **India’s population** | | | **Projected Covid-19 deaths** | | **India’s standardized population** | | | **Standardized Covid-19 deaths** | |
| --- | --- | --- | --- | --- | --- | --- | --- | --- | --- | --- | --- | --- | --- | --- | --- | --- |
|  | **Female** | **Male** | **Female** | **Male** | **Female** | **Male** | **Female** | | **Male** | **Female** | **Male** | **Female** | | **Male** | **Female** | **Male** |
| **Unit** | **persons** | | **per million** | | **per million** | | **thousand** | | | **deaths** | | **thousand** | | | **deaths** | |
| **Source** | **IR sample** | | **Kerala sample** | |  | | **United Nations** | | |  | |  | | |  | |
| 1 | 2 | 3 | 4 | 5 | 6 | 7 | 8 | 9 | | 10 | 11 | 12 | | 13 | 14 | 15 |
| **Age group** |  |  |  |  |  |  |  |  | |  |  |  | |  |  |  |
| **0-4** |  |  |  |  | *11* | *8* | 61,228 | 55,651 | | 429 | 521 | 58,160 | | 61,864 | 612 | 504 |
| **5-9** |  |  |  |  | *15* | *12* | 61,877 | 56,105 | | 647 | 628 | 56,921 | | 60,713 | 827 | 712 |
| **10-14** |  |  |  |  | *21* | *18* | 66,303 | 59,853 | | 1113 | 891 | 54,842 | | 58,689 | 1,174 | 1,073 |
| **15-19** |  |  |  |  | *33* | *30* | 66,671 | 59,375 | | 1912 | 1287 | 52,325 | | 56,059 | 1,741 | 1,709 |
| **20-24** | 4,156 | 48,356 | 22.4 | 22.3 | 54 | 54 | 64,866 | 57,639 | | 3338 | 1960 | 51,183 | | 54,580 | 2,767 | 2,929 |
| **25-29** | 6,513 | 75,781 | 37.7 | 40.8 | 91 | 98 | 62,039 | 55,358 | | 5936 | 3135 | 51,100 | | 54,185 | 4,633 | 5,327 |
| **30-34** | 8,870 | 103,207 | 64.5 | 76.8 | 155 | 185 | 58,875 | 53,301 | | 10,718 | 5251 | 52,456 | | 54,748 | 8,151 | 10,122 |
| **35-39** | 11,227 | 130,632 | 111.7 | 146.2 | 269 | 352 | 53,994 | 49,466 | | 18,884 | 8721 | 47,513 | | 48,943 | 12,785 | 17,233 |
| **40-44** | 13,583 | 158,058 | 193.4 | 278.1 | 466 | 670 | 46,631 | 43,589 | | 31,234 | 13,929 | 43,269 | | 44,152 | 20,149 | 29,572 |
| **45-49** | 15,940 | 185,483 | 330.8 | 521.2 | 797 | 1255 | 40,757 | 38,684 | | 51,442 | 22,340 | 42,160 | | 42,708 | 33,586 | 53,601 |
| **50-54** | 18,297 | 212,909 | 553.3 | 949.0 | 1332 | 2285 | 35,203 | 33,673 | | 81,318 | 34,498 | 39,509 | | 39,411 | 52,645 | 90,066 |
| **55-59** | 20,654 | 240,334 | 895.0 | 1656.2 | 2155 | 3988 | 30,150 | 29,106 | | 122,195 | 51,126 | 34,635 | | 34,030 | 74,649 | 135,723 |
| **60-64** |  |  |  |  | *3335* | *6580* | 24,695 | 24,196 | | 166,174 | 69,339 | 29,205 | | 27,827 | 97,385 | 183,112 |
| **65-69** |  |  |  |  | *4881* | *10,123* | 19,168 | 19,092 | | 200,032 | 83,618 | 24,911 | | 22,828 | 121,579 | 231,096 |
| **70-74** |  |  |  |  | *6684* | *14,325* | 11,628 | 12,464 | | 173,510 | 76,950 | 17,968 | | 15,436 | 120,108 | 221,111 |
| **75-79** |  |  |  |  | *8473* | *18,388* | 7,063 | 8,021 | | 137,149 | 63,386 | 12,221 | | 9,694 | 103,547 | 178,257 |
| **80+** |  |  |  |  | *10,211* | *21,621* | 5,953 | 7,332 | | 139,781 | 67,680 | 15,857 | | 9,903 | 161,913 | 214,111 |
| **Population by sex** | 99,240 | 1,154,760 |  |  |  |  | 717,101 | 662,905 | |  |  | 684,236 | | 695,770 |  |  |
| **Deaths by sex** |  |  | 9 | 247 | 1880 | 72 |  |  | | 593,108 | 1,108,728 |  | |  | 818,253 | 1,376,256 |
| **Total deaths** |  |  | 256 | |  | |  |  | | **1,701,836** | |  |  | | **2,194,510** | |
| **Covid-19 death rate (per 1000)** | | |  |  |  |  |  | Crude rate | | | 1.20 |  | Standardized death rate | | | 1.6 |

Columns:

2-3: IR workforce age and sex distribution

4-5: Covid-19 death rates by age and sex derived from the Kerala sample.

6-7: Adjusted from preceding columns to fit the observed death among the IR sample (*rates in italics* used only for India’s population)

8-9: Age and sex distribution of India’s population in 2020 (United Nations estimates) [52]

10-11: Projected Covid-19 deaths on May 7 computed from columns 6-7 and 8-9

12-13: Age and sex distribution of India’s population standardized by the world’s population (United Nations estimates)

14-15: Standardized Covid-19 deaths computed from columns 6-7 and 12-13
