# Supplementary material for: An alternative estimation of the death toll of the Covid-19 pandemic in India
Source: PLoS One. 2022 Feb 16;17(2):e0263187. doi: 10.1371/journal.pone.0263187 (PMC8849468; doi:10.1371/journal.pone.0263187)
Supplement: S1 Table — (DOCX) [file pone.0263187.s003.docx]

**Supplementary table S1: Population, Covid-19 deaths and Covid-19 death rates, Kerala, India, 2021.**

| **Kerala** | **Population** | | **Covid-19**  **Deaths** | | **Covid-19**  **Death rates** | |
| --- | --- | --- | --- | --- | --- | --- |
| **All ages** | **Male** | **Female** | **Male** | **Female** | **Male** | **Female** |
| **0-4** | 1,181 | 1,134 | 13 | 16 | 11 | 14 |
| **5-9** | 1,234 | 1,182 | 3 | 1 | 2 | 1 |
| **10-14** | 1,283 | 1,233 | 10 | 5 | 8 | 4 |
| **15-19** | 1,316 | 1,255 | 15 | 16 | 11 | 13 |
| **20-24** | 1,363 | 1,284 | 31 | 35 | 23 | 27 |
| **25-29** | 1,329 | 1,292 | 67 | 52 | 50 | 40 |
| **30-34** | 1,261 | 1,337 | 141 | 97 | 112 | 73 |
| **35-39** | 1,186 | 1,358 | 280 | 171 | 236 | 126 |
| **40-44** | 1,127 | 1,352 | 456 | 255 | 405 | 189 |
| **45-49** | 1,102 | 1,343 | 707 | 406 | 642 | 302 |
| **50-54** | 1,067 | 1,282 | 1,064 | 651 | 997 | 508 |
| **55-59** | 983 | 1,153 | 1,406 | 972 | 1,431 | 843 |
| **60-64** | 849 | 974 | 1,988 | 1,271 | 2,342 | 1,305 |
| **65-69** | 696 | 795 | 2,257 | 1,476 | 3,243 | 1,857 |
| **70-74** | 505 | 617 | 2,428 | 1,595 | 4,808 | 2,584 |
| **75-79** | 305 | 424 | 1,884 | 1,372 | 6,178 | 3,235 |
| **80+** | 256 | 432 | 2,821 | 2,665 | 11,021 | 6,168 |
| **Total** | 17,043 | 18,447 | 15,572 | 11,056 | 914 | 599 |
| **Unit** | 000s | |  | | per 1,000,000 | |
| **Source** | <https://main.mohfw.gov.in/reports-0> | | <https://dashboard.kerala.gov.in/covid/deaths.php> | | computed by the author | |
| **Date** | 2021 | | 18/10/21 | | 18/10/21 | |
